# Role of NO-sensitive guanylyl cyclase in angiogenesis and arteriogenesis

**DOI:** 10.1186/2050-6511-14-S1-P8

**Published:** 2013-08-29

**Authors:** Noomen Bettaga, Andreas Friebe, Ronald Jäger

**Affiliations:** 1 Institut für Physiologie, Julius-Maximilians-Universität, Würzburg, Germany

## Background

As the main receptor for nitric oxide (NO), NO-sensitive guanylyl cyclase (NO-GC) is involved in the regulation of different physiological processes such as the regulation of blood pressure. cGMP synthesis increases upon NO generation by the endothelial NO synthase (eNOS) and mediates vascular smooth muscle relaxation. NO synthesis can be stimulated by the vascular endothelial growth factor (VEGF) which is an important stimulator of angiogenesis. The interconnection between the VEGF and the NO/cGMP pathways is still unclear. In this project, we investigated the role of NO/cGMP signaling in angiogenesis and arteriogenesis using NO-GC-deficient mice.

**Figure 1:**
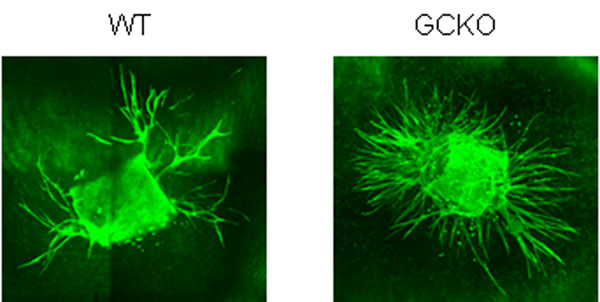
VEGF-induced sprouting of aortae from WT and GCKO mice.

**Figure 2:**
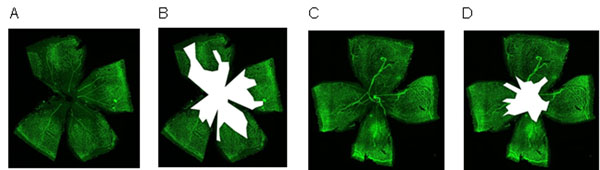
Vascularization of the retina at different stages of oxygen-induced retinopathy. A) WT after hyperoxia; B) WT, quantified avascular area; C) GCKO after hyperoxia; D) GCKO, quantified avascular area

To investigate VEGF-mediated angiogenesis, the aortic ring assay was employed. Endothelial sprouting was measured in aortic rings from global NO-GC knockout mice (GCKO) and WT animals (see Figure 1). We also used the oxygen-induced retinopathy model (OIR) to monitor vessel loss and regrowth *in vivo* dependent on the presence of NO-GC (see figure 2). To determine a possible role of NO-GC in arteriogenesis, we used the hindlimb ischemia model applied on GCKO and smooth muscle cell-specific knockout mice.

## Results

Our results show differences between GCKO and WT animals in aortic ring assay, OIR and hindlimb ischemia experiments. In the aortic ring assay, VEGF-induced sprouting was stronger in GCKO than in WT mice. Oxygen-induced retinopathy experiments revealed smaller avascular areas and higher tuft growth in GCKO compared to WT mice. Hindlimb ischemia experiments show delayed reperfusion in GCKO with respect to WT animals.

## Conclusion

Our data indicate the involvement of NO-sensitive guanylyl cyclase in the regulation of angiogenesis as NO-GC has an inhibitory effect on this VEGF-induced process. Preliminary data of hindlimb ischemia indicate a prominent role of NO-GC in arteriogenesis.

